# Validation of endothelin-1 and interleukin-1β as a biomarker for diagnosing peri-implant disorders

**DOI:** 10.6026/9732063002001148

**Published:** 2024-09-30

**Authors:** Pranav V Manek, Arpita Srivastava, Rahul Shrivastava, Miloni Bhatt, Naina Pattnaik, Manish Kumar

**Affiliations:** 1 Department of Oral Medicine and Radiology, Pacific Dental College and Research Centre, Udaipur, Rajasthan, India; 2 Department of Oral Medicine and Radiology, Government College of Dentistry Indore, M.P, India; 3 Private Practitioner, Department of Prosthodontics, Revti Dental Clinic, Indore, MP, India; 4 Department of Prosthodontics, Karnavati school of dentistry, Karnavati University, Gujarat, India; 5 Department of Periodontics and Oral Implantology, Kalinga Institute of Dental Science, KIIT Deemed to be University Patia, Bhubaneswar, Odisha, India; 6 Department of Dentistry, Dr. Laxminarayan Pandey Government Medical College & Hospital, Ratlam, M.P, India

**Keywords:** Endothelin-1, biomarker, peri-implant mucositis, peri-implantitis

## Abstract

Endothelin-1 (ET-1) and interleukin-1β (IL-1β) is increased in periodontitis and is linked to inflammatory cytokines among other variables. The purpose of this study was to ascertain if ET-1 and IL-1β are utilized as an early indicator for peri-implant mucositis (PM) and peri-implantitis (PI), as well as to look into the relationship between ET-1 and IL-1β levels and peri-implant illnesses. 58 patients (30 males and 28 females) with a total of 152 implants were included for final analyses. Three groups were formed from the 152 qualifying implants. A plastic probe was used at low pressure of 0.25N to assess the peri-implant parameters like probing pocket depth (PPD), modified gingival index (mGI), BOP being present or absent and modified plaque index (mPI). Mean bone loss (MBL) and the average annual bone loss (ABL) were evaluated. The values were significantly greater in PI group and PM group as compared to healthy subjects. ET-1 and IL-1β levels are significantly increased in peri-implant illnesses. ET-1 and IL-1β may be utilized as diagnostic indicator for peri-implant disorders.

## Background:

Reliable non-invasive methods for the early diagnosis of peri-implant disorders have emerged in the form of biomarker-based diagnostic methodologies [1, 2, 3]. Anticipatory detection or the identification of biomarkers linked to degradation of peri-implant tissue before clinical symptoms manifest, is made possible [4, 5, 6, 7]. As a result, their combination with traditional techniques can increase the precision of early identification and disease advancement prediction [8, 9, 10, 11, 12]. Biomarkers such as Matrix metalloproteinase 8 (MMP-8) and Interleukin 1β (IL-1β) have been found to be useful for identifying peri-implantitis within the peri-implant sulcus fluid (PISF) by some research [13, 14, 15]. Since peptides have a lower molecular weight than inflammatory cytokines, they are relatively vascular-permeable. Peptides that escape from surrounding tissues as a result of vasodilatation brought on by inflammation therefore have potential for the early identification of peri-implant disorders [16, 17, 18].

Dental implants have been more popular as a prosthetic means of treatment in the past few years; however the rise in peri-implant infections has caused some concern. Peri-implant mucositis (PM) and peri-implantitis (PI) are the two categories into which these conditions can be divided [1-14]. The prevalence figures of PM vary from 19% to 69 % and while prevalence of PI varies from 1% to 40%. A transitory soft tissue inflammatory condition surrounding implants with no any degradation of supporting bone or on-going marginal bone degeneration is known as PI, a plaque-induced illness [13-16].

Although the clinical recognition is complicated and its progression to peri-implantitis remains uncertain, it is thought to be a pathological prior to peri-implantitis [13-14]. Peri-implant illnesses are usually treated with a mix of non-invasive, invasive, and pharmaceutical treatments [11-14]. There is disagreement over the most successful therapy, even though another research reported a forty-two percent cure frequency at 5 years and numerous comparable procedures are available [12-15]. In light of these factors, it is imperative to accurately diagnose PM in order to lower the possibility of PI [16-18]. A classification approach for peri-implant illnesses and disorders was presented at the 2017 World Workshop [11-14]. The principal means of diagnosis in this classification approach is clinical evaluations like bleeding on probing (BOP) and pocket probing depths (PPD) including radiographic image evaluation [14, 16]. Nevertheless, peri-implant disease progression, prospective loss of crestal bone, and failure of the implants cannot be well predicted by these parameters alone [18-22]. For example, the binary existence or absence of a single variable determines BOP. Because peri-implant mucosal connection is sensitive, low probing pressure (about 0.25 N) is necessary to prevent excessive depths of probing [16, 20]. Furthermore, because of the limited probing directions, massive over-contoured implant architecture can result in severe BOP and hemorrhage [19-24]. As a result, a minimally invasive diagnostic method that can precisely ascertain the peri-implant state is needed [10- 17].

Vascular endothelial cells emit a 21-amino acid peptide called endothelin-1 (ET-1), which was first discovered in 1988 [21-24]. ET-1 has a variety of regulatory functions. With a molecular weight of only about 2.5 kDa, it exerts strong vasoconstrictor action on a number of physiological functions and may slow the development of inflammatory disorders and hypertension [25-27]. According to research, people with periodontitis had much greater ET-1 levels in their gingival sulcus exudate (GSE) than healthy GSE [14-18]. According to another research, ET-1 controls the expression of IL-1β in gingival tissues [12-17]. Although its precise effects are unclear, ET-1 is increased in periodontitis and is linked to inflammatory cytokines among other variables [18-23]. Despite the differences in the supporting tissue architecture between periodontitis and peri-implantitis, many clinical characteristics and indicators are similar [21-27]. ET-1's function in PM and PI hasn't been studied, though. Thus, the purpose of this study was to ascertain if ET-1 and IL-1β may be utilized as an early indicator for PM and PI, as well as to look into the relationship between ET-1 and IL-1β levels and peri-implant illnesses.

## Methods and Materials:

## Study design:

We enlisted a cohort of 124 patients-a total of 274 implants - which underwent dental implant maintenance therapy at a tertiary level dental hospital.

## The inclusion criteria were:

(1) Above the age of twenty

(2) Not being pregnant or nursing

(3) Utilizing operational implants for a minimum of a year

(4) Absence of a history of inadequately managed systemic illnesses

(5) No prior nonsurgical or surgical therapy history, including scaling at the location, to be evaluated within three months following the examination.

(6) No previous history of treatment during the three months prior to the examination as well as sampling.

## The following were the exclusion standards:

(1) Implants present but no prior radiography (base data),

(2) The existence of implants positioned so that probing was challenging or with superstructures

(3) The existence of implants with BOP (-) and an average marginal loss of bone ≥0.2 mm

(4) The existence of implants whose radiographic pictures were too hazy to quantify the quantity of bone resorption.

Ultimately, 58 patients (30 males and 28 females) with a total of 152 implants were included for final analyses.

## Clinical evaluations:

A qualified dentist (PQ) evaluated the implants of those participating in study applying the following metrics: A plastic probe was used at low pressure of 0.25N to assess the peri-implant parameters like PPD, modified gingival index (mGI), BOP being present or absent and modified plaque index (mPI). Every evaluator (AB,AC and AD) received a randomly selected set of radiographs for the radiographic evaluation. The evaluators were unaware of any details that may be used to identify the participant. The distance that exists between the implant's most apical aspect and junction point of implant with proximal bone was measured to assess the bone loss around implants. It was carried out by applying software for measuring distance in an electron microscope during radiographic imaging. The implant shoulder functioning as the reference position. The mean of the three evaluators' calculation was then computed. Mean bone loss (MBL) was determined after modifying values corresponding to magnification ratio of the length of the implant body. The average annual bone loss (ABL) surrounding the implants was then computed and related with the MBL at baseline.

## Patient groups:

Three groups were formed from the 152 qualifying implants, in accordance with the guidelines provided by the 2017 World Workshop [10-14] described by the attributes listed below:

Group 1: Healthy (n = 58)

Group 2: Peri-implant Mucositis (PM) (n = 44)

Group 3: Peri-implantitis (PI) (n = 50)

The Toronto conference [4] defined pathological bone resorption as an ABL of ≥0.2 mm at the peri-implant region.

## Healthy group:

Implants that show no other indications of signs of inflammation on the oral mucosa, ABL<0.2 mm and BOP(-).

## PM group:

Implants with ABL<0.2 mm and BOP (+).

## PI group:

Implants with ABL > 0.2 mm and BOP (+).

## PISF sampling:

Plaque above the peri-implant margin was removed using plastic curettes. Cotton rolls were used to separate the sampling locations and a light breeze was used to dry them. After being carefully introduced <1-2 mm into the deepest sulcus until a minor resistance was felt, PerioPaper was maintained in situ for one minute. The same procedure was used to gather samples from the same location five times, with a one-minute break in between. After ten minutes, any PerioPaper that had come into contact with either saliva or blood was thrown away and replaced. Using a Periotron 8000 instrument, volume quantification was done right away following sample collection in order to reduce evaporation. As directed by the manufacturer, the Periotron 8000 had been calibrated before the study and then recalibrated on a regular basis. The volume of PISF (µL) for periotron values is stated in relation to the appropriate validated logarithmic curve [25]. PerioPaper was kept in plastic sealable Eppendorf tubes, frozen at -80 °C until analysis, in a 50 µL combination of protease inhibitors and phosphate-buffered saline (PBS).

## Enzyme-linked immunosorbent assay:

After being collected by PerioPaper, the solution was centrifuged for 10 minutes at 4°C at 300 rpm. Centrifugation was then run for two minutes at 12,000 rpm. After collecting the ensuing supernatant, five supernatants were mixed together to create a volume of 250 µL. The ELISA Endothelin-1 Immunoassay kit was used to assess ET-1 levels, and the ELISA Huma IL-1β/IL-1F2 kit was used to measure IL-1β levels. The ELISA protocols were carried out in compliance with the manufacturer's guidelines. Sites exhibiting cytokine amounts below the assay's detection limit were noted as 0. The concentrations of these biomarkers were represented as ET-1 (ρg/site) and IL-1β (µg/site), after being adjusted for the quantity of PISF [26].

## Statistical analysis:

Sample size estimates were performed using G*Power 3.1.9.6 software, with an effect size of 0.8, a two-tailed significance level of 95% (α < 0.05) and statistical power of 80%. Based on these criteria, the study's sample size need was 42 implants minimum per group in order to identify any variations between the groups. Excel's Bell Curve was used to do statistical studies. The Shapiro-Wilk test was used to assess the normality of the data. For each independent variable, the statistically significant differences between groups were found using the Kruskal-Wallis test, followed by a Steel-Dwass adjustment. ROC curve analysis and area under the ROC curve (AUC) were used to assess the diagnostic reliability of the biomarker choices for differentiating between peri-implant mucositis or peri-implantitis and healthy implants. Every biomarker was modified, as was its corresponding logistic regression model (which took into account the dental implant's age and sex). The best cut-offs from the ROC curves for each biomarker (unadjusted and adjusted models) was found using the Youden index. To evaluate the quality of categorization, diagnostic sensitivity and specificity were computed for every biomarker utilizing a cut-off value. At p<0.05, statistical significance was established.

## Results:

The demographic details of implants across the three groups are presented in Table 1. The mean age of participants with healthy implants, implants with PM, and implants with PI were 70.8±7.5 years, 74.9±8.8 years and 68.9±8.4 years, respectively. The proportion of females was higher in the healthy implant and PI groups compared to the PM group. In all three groups, the mandible was the most common site for implant placement, and the molar region was the most frequent area for implant placement (Table 1).

BOP was positive in all patients of implants with PM group and implants with PI. ABL was maximum (0.43±0.004 mm) in PI group as compared to healthy (0.04±0.002 mm) and PM group (0.06±0.001 mm). The ABL was comparable in healthy and PM group. PPD was maximum in PI group (5.3±0.02 mm) followed by PM group (4.2±0.07 mm) and healthy group (3.1± 0.04 mm). The values of mean mPI and mGI were greater in PI group and PM group compared to healthy subjects. However, the values in PI group were comparable to values in PM group (Table 2).

PISF volume was 1.56 (0.90-2.81) µL in healthy subjects, 3.39 (2.87- 4.56) µL in PM group and 4.26 (2.40-5.24) µL in PI group. The values of PISF volume was significantly greater in PI group and PM group as compared to healthy group (p<0.001). However, the values in PI group and PM groups were comparable (Table 3).

The values of ET-1 was 0.18 x 10-3 (0.08 x 10-3 - 0.62 x 10-3) ρg/site in healthy subjects, 1.03 x 10-3(0.35 x 10-3 - 2.76 x 10-3) ρg/site in PM group and 0.48 x 10- 3 (0.27 x 10- 3 - 0.98 x 10- 3) ρg/site in PI group. The values were significantly greater in PI group and PM group as compared to healthy subjects (Table 4). The values of IL-1β was 0.04 (0.02-0.09) µg/site in healthy subjects, 0.16 (0.10-0.31) in PM group and 0.09 (0.03-0.52) in PI group. The values were significantly greater in PI group and PM group as compared to healthy subjects (Table 4).

The overall sensitivity and specificity of ET-1 as biomarker according to univariate analysis in differentiating PI and healthy subjects was 93% and 57% respectively. The findings were statistically significant (p value < 0.01), 95% CI (0.59-0.87). The overall sensitivity and specificity of IL-1β as biomarker according to univariate analysis in differentiating PI and healthy subjects was 65% and 60% respectively. The findings were statistically significant (p value < 0.01), 95% CI (0.57-0.85).

The overall sensitivity and specificity of ET-1 as biomarker according to adjusted analysis in differentiating PI and healthy subjects was 81% and 80% respectively. The findings were statistically significant (p value < 0.01), 95% CI (0.64-0.90). The overall sensitivity and specificity of IL-1β as biomarker according to adjusted analysis in differentiating PI and healthy subjects was 65% and 67% respectively. The findings were statistically significant (p value < 0.01), 95% CI (0.55-0.84) (Table 5).

The overall sensitivity and specificity of ET-1 as biomarker according to univariate analysis in differentiating PI and healthy subjects was 64% and 84% respectively. The findings were statistically significant (p value < 0.01), 95% CI (0.64-0.90). The overall sensitivity and specificity of IL-1β as biomarker according to univariate analysis in differentiating PI and healthy subjects was 60% and 77% respectively. The findings were statistically significant (p value < 0.01), 95% CI (0.55-0.85). The overall sensitivity and specificity of ET-1 as biomarker according to adjusted analysis in differentiating PI and healthy subjects was 69% and 70% respectively. The findings were statistically significant (p value < 0.01), 95% CI (0.62-0.89). The overall sensitivity and specificity of IL-1β as biomarker according to adjusted analysis in differentiating PI and healthy subjects was 60% and 77% respectively. The findings were statistically non-significant (p value=0.06), 95% CI (0.50-0.82) (Table 6).

## Discussion:

In recent years, dental implants have become more and more common as a prosthetic treatment option; however, the rise in peri-implant infections, or PM and PI, has raised some concerns [14-17]. PM and PI are two categories into which these conditions can be divided; prevalence figures for PM range from 19 to 65 percent, while those for PI range from 1 to 47 percent [1-7]. PI is a plaque-induced illness that is a transient soft tissue inflammatory condition that surrounds implants without any degradation of the supporting bone or on-going marginal bone degeneration [10-16]. The purpose of this study was to ascertain if ET-1 and IL-1β may be utilized as an early indicator for PM and PI, as well as to look into the relationship between ET-1 and IL-1β levels and peri-implant illnesses. After carrying out univariate analysis and adjusted analysis, it was found that overall sensitivity of ET-1 and IL-1β in differentiating healthy subjects against PM and PI was in range 60% to 95%. Similarly, the overall specificity was between 60%-84%. These values suggest that ET-1 and IL-1β can be a diagnostic precursor for PM and PI.

The findings of present research are also supported by other research [23-27]. These research have also indicated about the possibility of ET-1 being used as diagnostic precursor for peri implant disorders [25-27].A study [17] found that gingival sulcus exudate (GSE) from periodontitis patients had significantly higher ET-1 levels than GSE from healthy subjects. Another study [18-21] claims that ET-1 regulates the expression of IL-1β in gingival tissues. ET-1 performs a number of regulatory tasks. Its molecular weight allows it to have a potent vasoconstrictor effect on several physiological processes and may delay the onset of inflammatory diseases and hypertension [15-19]. In our study the values of ET-1 were significantly greater in PI group and PM group as compared to healthy subjects. The values of IL-1β were significantly greater in PI group and PM group as compared to healthy subjects.

The findings of this study are similar to findings of other research [24 -27]. Some research has also reported increased concentrations of ET-1 and IL-1β in patients with peri-implant disorders like PI and PM [11-18]. ET-1 is elevated in periodontitis and is associated with inflammatory cytokines among other factors, albeit its exact effects remain unknown [21-25]. Many clinical features and indications of periodontitis and peri-implantitis are similar, despite the changes in the supporting tissue architecture. However, the role of ET-1 in PM and PI has not been investigated [20-25]. Biomarker-based diagnostic approaches have emerged as dependable non-invasive tools for the early detection of peri-implant diseases in some research [10-16]. This makes it possible to identify biomarkers associated with peri-implant tissue degeneration before clinical symptoms appear, a process known as anticipatory detection [13-17]. Consequently, their fusion with conventional methods can improve the accuracy of early detection and prognostication of disease progression according to other investigations [11-15]. Peptides are relatively vascular-permeable because they have a smaller molecular weight than inflammatory cytokines. Thus, peptides that escape from surrounding tissues due to vasodilatation induced by inflammation may be useful in the early detection of illnesses related to the period surrounding implants [21-27].

In our study PISF volume was 1.56 (0.90-2.81) µL in healthy subjects, 3.39 (2.87- 4.56) µL in PM group and 4.26 (2.40-5.24) µL in PI group. The values of PISF volume was significantly greater in PI group and PM group as compared to healthy group (p<0.001). However, the values in PI group and PM groups were comparable. Some studies have reported findings that are similar to those of our research [21-27]. Studies have values of PISF volume significantly greater in PI group and PM group as compared to healthy group [12- 18]. Studies indicate that biomarkers like Matrix metalloproteinase 8 (MMP-8) and Interleukin 1β (IL-1β) can be helpful in detecting peri-implantitis in the peri-implant sulcus fluid (PISF) [20- 24]. Since preliminary biomarker identification for peri-implant disorders is still challenging due to insufficient evidence of biomarkers with elevated concentrations in peri-implant mucositis, we focused on peptides with known regulatory consequences on inflammatory cytokines [21-26]. Biomarker-based diagnostic approaches have emerged as dependable non-invasive tools for the early detection of peri-implant diseases [9 -15]. This makes it possible to identify biomarkers associated with peri-implant tissue degeneration before clinical symptoms appear, a process known as anticipatory detection [11 -18]. Consequently, their fusion with conventional methods can improve the accuracy of early detection and prognostication of disease progression [10-16]. The observations of our study are consistent with those of other studies, which also demonstrated that peri-implant parameters such as PPD, PI, GI, and ABL were higher in cases of PI and PM [10-19]. Clinical evaluations such as bleeding on probing (BOP) and pocket probing depths (PPD), including radiographic image evaluation, are the primary means of diagnosis in this classification approach as suggested by some investigations [17, 18]. However, these characteristics alone are not a reliable indicator of the course of peri-implant illness, the potential loss of crestal bone, or the implant's failure according to some research [19- 24]. Low probing pressure (about 0.25 N) is required to avoid probing too deeply since the peri-implant mucosal connection is sensitive. Moreover, huge over-contoured implant architecture may produce severe bleeding and bleeding due to the limited probing orientations [16-23]. Therefore, there is a need for a minimally invasive diagnostic technique that can accurately determine the peri-implant condition.

## Conclusion:

ET-1 and IL-1β levels are significantly increased in peri-implant illnesses.ET-1 and IL-1β may be utilized as diagnostic indicator for peri-implant disorders.

## Figures and Tables

**Table 1:** Demographic details, location of the implants in three groups

	**Age (years)**	**Gender**		**Jaw of sampling site**		**Location in arch**		
	Mean± SD	Female n (%)	Male n (%)	Mandible n (%)	Maxilla n (%)	Incisor n (%)	Pre Molar n (%)	Molar n (%)
Healthy (n = 58)	70.8±7.5	24 (41.37)	34 (58.62)	34 (58.62)	24 (41.37)	18(31.03)	20 (34.48)	20(34.48)
Mucositis (n = 44)	74.9±8.8	24(54.44)	20 (45.45)	30 (68.18)	14 (31.81)	10 (22.72)	16 (36.36)	18 (40.90)
Peri-implantitis (n = 50)	68.9±8.4	22 (44.00)	28 (56.00)	36 (72)	14 (28)	20 (40)	8 (16)	22 (44)

**Table 2:** Data regarding clinical and radiographic variables in three groups

			**Peri-implant parameters**		
	BOP (+/-)	ABL(mm) mean± SD	PPD(mm) mean± SD	mPImean± SD	mGImean± SD
Healthy (n = 58)	(0/58)	0.04±0.002	3.1± 0.04	0.2±0.001	0.3±0.002
Mucositis (n = 44)	(44/0)	0.06±0.001	4.2±0.07	2.1±0.04	2.4±0.03
Peri-implantitis (n = 50)	(50/0)	0.43±0.004	5.3±0.02	2.1±0.03	2.4±0.05

**Table 3:** PISF volumes in three categories

	**PISF volumes (µL) Median (IQR)**	**P value**
Healthy (n = 58)	1.56 (0.90-2.81)	
Mucositis (n = 44)	3.39 (2.87- 4.56)	<0.001
Peri-implantitis (n = 50)	4.26 (2.40-5.24)	

**Table 4:** Comparison of biomarkers ET-1 and IL-1β in three groups

	**Values of ET-1 (ρg/site) Median (IQR)**	**P value**
Healthy (n = 58)	0.18 x 10^-3^ (0.08 x 10^-3^ - 0.62 x 10^-3^)	
Mucositis (n = 44)	1.03 x 10^-3^(0.35 x 10^-3^ - 2.76 x 10^-3^)	<0.001
Peri-implantitis (n = 50)	0.48 x 10^-3^ (0.27 x 10^-3^ - 0.98 x 10^-3^)	
	Values of IL-1β (µg/site) Median (IQR)	
Healthy (n = 58)	0.04 (0.02-0.09)	
Mucositis (n = 44)	0.16 (0.10-0.31)	<0.001
Peri-implantitis (n = 50)	0.09 (0.03-0.52)	

**Table 5:** Comparison of biomarkers ET-1 and IL-1β in three groups

	**Univariable model**		**Adjusted Model**	
Biomarker	ET-1	IL-1β	ET-1	IL-1β
Cut-off value	0.22	0.45	0.42	0.4
AUC value	0.73	0.71	0.77	0.7
95% Cl	0.59-0.87	0.57-0.85	0.64-0.90	0.55-0.84
p-value	< 0.01	< 0.01	< 0.01	0.01
Sensitivity	93	65	81	65
Specificity	57	60	80	67

**Table 6:** Comparison of healthy implants and peri-implant mucositis

	**Univariable model**		**Adjusted Model**	
Biomarker	ET-1	IL-1β	ET-1	IL-1β
Cut-off value	0.9	0.16	0.4	0.47
AUC value	0.77	0.7	0.76	0.66
95% Cl	0.64-0.90	0.55-0.85	0.62-0.89	0.50-0.82
p-value	< 0.01	0.01	< 0.01	0.06
Sensitivity	64	60	69	60
Specificity	84	77	70	77
